# TRIM59 Promotes the Proliferation and Migration of Non-Small Cell Lung Cancer Cells by Upregulating Cell Cycle Related Proteins

**DOI:** 10.1371/journal.pone.0142596

**Published:** 2015-11-24

**Authors:** Weihua Zhan, Tianyu Han, Chenfu Zhang, Caifeng Xie, Mingxi Gan, Keyu Deng, Mingui Fu, Jian-Bin Wang

**Affiliations:** 1 Institute of Translation Medicine, Nanchang University, Nanchang City, Jiangxi, 330031, China; 2 Department of Basic Medical Science, School of Medicine, University of Missouri Kansas City, Kansas City, MO, 64108, United States of America; Wayne State University School of Medicine, UNITED STATES

## Abstract

TRIM protein family is an evolutionarily conserved gene family implicated in a number of critical processes including inflammation, immunity, antiviral and cancer. In an effort to profile the expression patterns of TRIM superfamily in several non-small cell lung cancer (NSCLC) cell lines, we found that the expression of 10 TRIM genes including TRIM3, TRIM7, TRIM14, TRIM16, TRIM21, TRIM22, TRIM29, TRIM59, TRIM66 and TRIM70 was significantly upregulated in NSCLC cell lines compared with the normal human bronchial epithelial (HBE) cell line, whereas the expression of 7 other TRIM genes including TRIM4, TRIM9, TRIM36, TRIM46, TRIM54, TRIM67 and TRIM76 was significantly down-regulated in NSCLC cell lines compared with that in HBE cells. As TRIM59 has been reported to act as a proto-oncogene that affects both Ras and RB signal pathways in prostate cancer models, we here focused on the role of TRIM59 in the regulation of NSCLC cell proliferation and migration. We reported that TRIM59 protein was significantly increased in various NSCLC cell lines. SiRNA-induced knocking down of TRIM59 significantly inhibited the proliferation and migration of NSCLC cell lines by arresting cell cycle in G2 phase. Moreover, TRIM59 knocking down affected the expression of a number of cell cycle proteins including CDC25C and CDK1. Finally, we knocked down TRIM59 and found that p53 protein expression levels did not upregulate, so we proposed that TRIM59 may promote NSCLC cell growth through other pathways but not the p53 signaling pathway.

## Introduction

The tripartite motif (TRIM) protein family comprises over 70 members that are implicated in a wide array of cellular processes, including cell growth [1], differentiation [2], development [3], apoptosis [4], inflammation and immunity [5, 6]. The most striking feature of TRIM superfamily proteins is the highly conserved order of domains in the RBCC motif, which contains a RING domain, one or two B-box motifs and a coiled-coil region [7, 8]. Most TRIM proteins constitute a new class of single RING-finger E3 ubiquitin ligases that promote post-translational modifications of various substrates, including themselves [7]. The TRIM proteins also form multimerization by means of self-association via the coiled-coil domains, acting as scaffolds for assembly of multi-protein complexes that identify subcellular compartments [9]. Over the past decade, much attention has been garnered in exploring the role of TRIM proteins in innate immunity to viral infections [10]. In recent years, several groups reported that TRIM proteins are also acting as oncogenes or tumor suppressors implicating in various cancer growth. For example, Raheja et al. reported that TRIM3 is a bona fide tumor suppressor, its ability to inhibit cell proliferation depends on the NHL (named after the NCL1, HT2A and LIN41 repeat) domain and its RING domain [11]. TRIM16 inhibits neuroblastoma cell proliferation and migration in vivo and tumorigenicity in vitro through changes expression of cyclin D1 and p27 [12]. TRIM28 appears upregulated in many cancers. In early stage of lung tumors, high TRIM28 correlates with increased overall survival [1]. Moreover, TRIM28 reduces cell proliferation in model lung cancer cell lines by bridging HDAC1/E2F interactions [1]. Recently, Zhou et al (2014) reported that in gastric tumor, TRIM59 interacts with p53, promoting its ubiquitination and degradation; TRIM59 might promote gastric carcinogenesis via this mechanism [13].

In an effort to profile the expression pattern of TRIM superfamily in several NSCLC cell lines, the expression of several TRIM genes including TRIM3, TRIM7, TRIM14, TRIM16, TRIM21, TRIM22, TRIM29, TRIM59, TRIM66 and TRIM70 was significantly upregulated in NSCLC cell lines compared with normal human bronchial epithelial (HBE) cell line, whereas the expression of other TRIM genes including TRIM4, TRIM9, TRIM36, TRIM46, TRIM54, TRIM67 and TRIM76 was significantly down-regulated in NSCLC cell lines compared with that in HBE cells. As TRIM59 has been reported to act as a proto-oncogene that affects both Ras and RB signal pathways in prostate cancer models [14], we here focused on the role of TRIM59 in the regulation of NSCLC cell proliferation and migration. We first found that TRIM59 protein was significantly increased in various NSCLC cell lines. SiRNA-induced knocking down of TRIM59 significantly arrested the proliferation and migration of NSCLC cell lines by arresting cell cycle in G2 phase.

## Materials and Methods

### Cell culture

Human bronchial epithelial (HBE) cells were from Sciencell Company and grown in bronchial epithelial cell medium (ScienCell, 3211). Non-small cell lung cancer (NSCLC) cell lines (H1299, H292, A549) were from ATCC and SPC-A1 cell line was a gift from Dr. Xuerong Wang’s group (Department of Pharmacology, Nanjing Medical University). NSCLC cell lines were grown in RPMI 1640 medium (Gibco) supplemented with 10% fetal bovine serum (Gibco). All cells were cultured under an atmosphere of 5% CO_2_ at 37°C.

### RNA purification and Q-PCR analysis

Total RNA was extracted using TRIzol reagent (Invitrogen, 15596–026). The cDNA synthesis was performed using PrimeScript RT reagent kit with gDNA Eraser (Takara, RR047A). Q-PCR experiments were conducted using SYBR Green Premix Ex Taq II kit (Takara, RR820A) and RT-PCR System-Applied Bio-system. The relative amount of mRNA expression of target genes was calculated by the comparative Ct method using GAPDH as a control. All Q-PCR reactions were performed in triplicate. Data was acquired using ABI ViiATM 7 Real-Time PCR System instrument. All primers for the 72 TRIM genes were validated using universal cDNA standards (BD Clontech). Quantification was performed by ΔCt method, with 18S or actin used for normalization. mRNA with cycle times ≥ 34 were determined to be undetected. Normalized mRNA levels are expressed as arbitrary units by transformed the cycle times using 2^ΔCt^. The data were opposed to log2 and organized in a heat map using MEV software (Dana-Farber Cancer Institute, http://www.tm4.org/mev.html).

### Low serum assay and saturation density assay

For low serum assay, cells were plated at a density of 10^5^ cells in 12-well dishes and allowed to adhere overnight in RPMI 1640 supplemented with 10% FBS. On the following day, the cell number was counted as the data of day 0, the medium was changed to RPMI 1640 supplemented with 1% FBS and then changed every other day for 6 days. At the indicated times, the cells were trypsinized and counted with a hemocytometer. For saturation density assay, 10^5^ cells were seeded in 12-well plates in RPMI 1640 supplemented with 10% FBS. The medium was changed every other day. The cell density was determined by counting the cells on the sixth day.

### Colony formation assay

Colony formation assay was carried out with H1299 cells that were grown in RPMI 1640 with 10% FBS in 60 mm plates and transiently transfected with control siRNA, TRIM59 siRNA-1, TRIM59 siRNA-2 or untreated as a positive control. After transfection, the cells were trypsinized, counted and 500 cells were seeded in 6-well dishes, and allowed to adhere overnight. On the following day, the media was changed to RPMI 1640 + 5% FBS. All cells were then grown for 2 weeks, with medium changed every second day. Plates were fixed with 4% formaldehyde and stained with 2% crystal violet. The images were obtained by a digital camera.

### SiRNA

The knocking down of TRIM59 was carried out using two distinct Stealth Select RNAi duplexes (Life Technologies Company). Targeted oligonucleotide sequences were: siRNA-1: GCCUCUCUAUCUGUUUACCAAAGUU; siRNA-2: UCCUCGUGUACUGCCAUGCUCUCAU; Stealth RNAi negative control duplex from Life Technologies Company was used as a control. The RNAi nucletides were transiently transfected in either HBE cells or NSCLC cells using SuperFectin siRNA Transfection Reagent (Pufei, Shanghai, 2103–100) and the relative knock down efficiency was determined by TRIM59 antibody.

### Scratch wound healing assay and transwell migration assay

Cell migration was measured using a scratch assay [15]. H1299 cells were plated in 6-well plates to create a confluent monolayer of 90–100% confluence, then the monolayer was scraped in a straight line to create a “scratch” by a 200 μl pipette tip. After removing debris and adding fresh media containing 2% FBS, cells were photographed using phase contrast microscope (Olympus IX71; magnification: 200 ×; objective: LCAch20XPh; no filter; camera: DP72; exposure and image analysis: cellSens software; pixel size: 1.4 megapixel monochrome CCD). The migration distance was assessed using image J software (National Institutes of Health, http://rsb.info.nih.gov/ij/download.html). A migration rate was calculated by cell relative migration area for each treatment.

Transwell migration assay was performed using 8μm pore size transwell chambers (BD Falcon). A total of 10^5^ cells in 0.2 ml media supplemented with 1% FBS were plated in the upper chamber. The lower chamber of the transwell device was filled with 500 μl RPMI 1640 supplemented with 10% FBS. After incubation at 37°C for 10 hrs, cells remaining on the upper surface of the membrane were removed. The cells on the lower surface of the membrane were fixed, stained with crystal violet and then counted under a light microscope (Olympus IX71; magnification: 40 ×; objective: UplanFl4XPh; no filter; camera: DP72; exposure and image analysis: cellSens software; pixel size: 1.4 megapixel monochrome CCD). Total 400 cells were imaged.

### Cell cycle analysis

Freshly prepared cells were harvested and re-suspended in 0.5ml PBS. Then the cells were fixed with 70% alcohol on ice for at least 2 hrs. After centrifugation, the supernatant was discarded and sediment was washed with PBS for once. Cell pellet was re-suspended in 5ml PBS and then cells were counted. Re-suspended 2×10^5^ cells with 400μl guava cell cycle reagent (Millipore, 4700–0160). After incubating in water bath at 37°C for 15 min, cell cycle was analyzed by the Millipore Guava easyCyte™ flow cytometer (Millipore).

### Immunofluorescence staining

Cells were plated in 24-well plates and allowed to grow for 18–24 hours. Cells were fixed with chilled methanol for 5 min at room temperature and rinsed by PBS for three times. Then cells were blocked with 5% normal goat serum, 0.3% Triton X-100 in PBS at room temperature for 1 hour. Anti-PCNA antibody (Abcam, ab92552) incubation was performed for 2 hours at room temperature. After washing with PBS, rhodamine-conjugated goat anti-rabbit antibody (Proteintech, SA00007-2) was applied at room temperature for 1 hour in a dark place. Cells were washed with PBS for three times and then mounted with DAPI Fluoromount-G mounting medium (Southern Biotech, 0100–20). Finally, cells were photographed with a fluorescence microscopy (Olympus IX83; magnification: 200 ×; objective: LUCPlanFl20XPh; filter setting and timing: DAPI: BA420 filter and 220ms, PCNA: BA590 filter and 360ms; camera: DP80; exposure and image analysis: cellSens software; pixel size: 12.5 megapixel color CCD). Total 600 cells were imaged.

### Protein isolation and western blot

The proteins from tissues and cells were separated by standard 10% SDS-PAGE followed by transfering the proteins to a PVDF membrane. The proteins were detected by the following primary antibodies: TRIM59 (Sigma, R06835), cyclin B1 (Proteintech, 55004-1-AP), CDC25C (Proteintech, 16485-1-AP), CDK1 (Proteintech, 19532-1-AP), p53 (Proteintech, 10442-1-AP), PCNA (Abcam, ab92552) and β-actin (Santa Cruz, sc-4778) and followed by incubation with a secondary antibody. Staining was performed with ECL western blot detection reagent. Antibody to β-actin was served as the endogenous control. All experiments were performed in triplicate.

### Statistical analysis

For each experiment, three independent replicates were performed. All the data were expressed as mean ± SD. Statistical evaluation was conducted using the Student t test. The intergroup difference was compared by using one-way analysis of variance followed by Dunnett’s test. A p-value of less than 0.05 was considered statistically significant. *, *p*<0.05 vs. control; **, *p*<0.01 vs. control; ***, *p*<0.001 vs. control.

## Results

### TRIM59 is highly expressed in NSCLC cell lines

**Fig 1:**
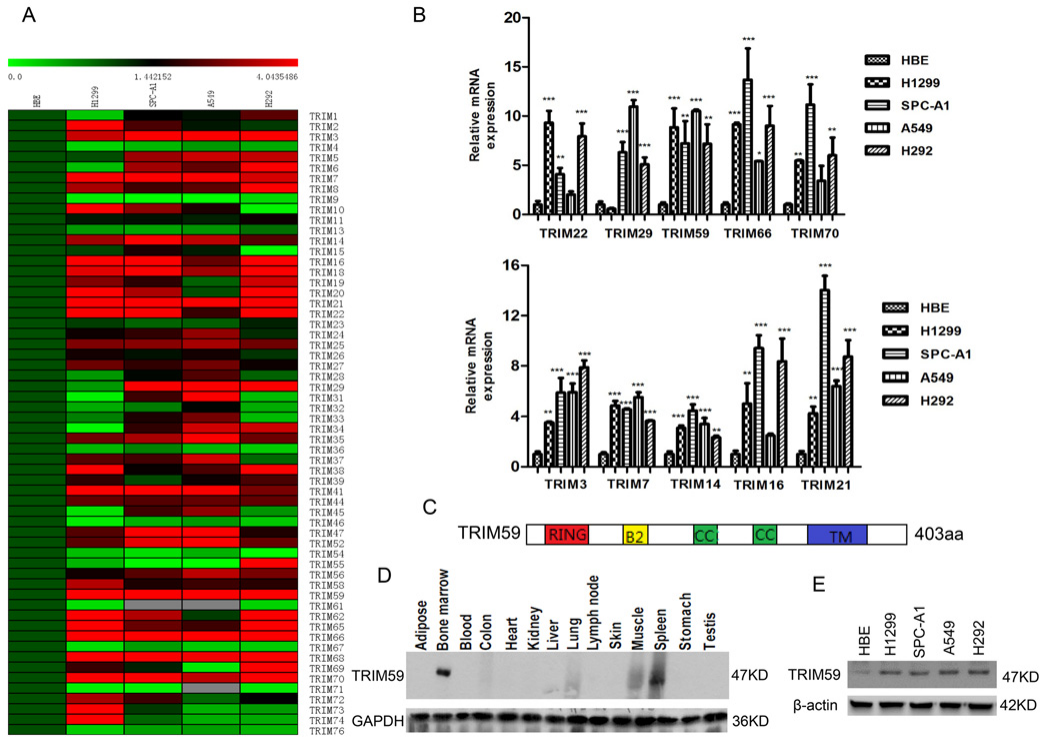
TRIM59 is highly expressed in NSCLC cell lines. **(A)** The mRNA expression levels of 60 TRIM family genes in four NSCLC cell lines (H1299, H292, SPC-A1 and A549) and normal human bronchial epithelial (HBE) cell line were determined by Q-PCR. The data were organized in a heat map by using MEV software. The relative expression levels of the genes were shown in the color scale of 0–4.0435486 in green-red-black color scheme. **(B)** The mRNA expression levels of TRIM genes including TRIM3, TRIM7, TRIM14, TRIM16, TRIM21, TRIM22, TRIM29, TRIM59, TRIM66 and TRIM70 were significantly upregulated in NSCLC cell lines compared with that in HBE cells. **(C)** Schematic depiction of TRIM59. TRIM59 contains a RING finger domain (RING), a B-box2 domain (B2), two coiled-coil domains (CC) and a transmembrane domain (TM). **(D)** The expression of TRIM59 in 14 kinds of normal tissues was checked by western blot using TRIM59 antibody. **(E)** The expression of TRIM59 protein in four NSCLC cell lines and HBE cell line. Lysates from the cell lines were subjected to immunoblot analysis with TRIM59 antibody.

Lung cancer is the most common cancer among the men worldwide. The main primary types of lung cancer are small cell lung carcinoma (SCLC) and non-small cell lung carcinoma (NSCLC). NSCLC is not well response to chemotherapy and has higher mortality than SCLC [16]. In an effort to profile the expression patterns of TRIM gene family in NSCLC cell lines, we selected four NSCLC cell lines including H1299, SPC-A1, A549 and H292. The human bronchial epithelial cell (HBE) was served as the control. The mRNA levels of TRIM gene family in these cell lines were measured by Q-PCR and analyzed by MEV software (S1 Table). The expression of several TRIM genes including TRIM3, TRIM7, TRIM14, TRIM16, TRIM21, TRIM22, TRIM29, TRIM59, TRIM66 and TRIM70 was significantly upregulated in NSCLC cell lines compared with HBE cells(Fig 1A and 1B), whereas the expression of other TRIM genes including TRIM4, TRIM9, TRIM36, TRIM46, TRIM54, TRIM67 and TRIM76 was significantly down-regulated in NSCLC cell lines compared with that in HBE cells (Fig 1A and S1 Fig). The mRNA expression levels of other 12 TRIM genes were not detected either in NSCLC cell lines or HBE cells. As TRIM59 has been reported to act as a proto-oncogene that affects both Ras and RB signal pathways in prostate cancer models [14], we here focused on TRIM59 in NSCLC for further research.

Human TRIM59 is a protein of 403aa containing a RING domain, a B-box domain, two coiled-coil domain and a transmembrane domain at its C-terminal (Fig 1C). Western blot showed that TRIM59 was highly enriched in bone marrow and spleen, low level in muscle and lung, undetectable in other tissues that were examined (Fig 1D). In addition, western blot further confirmed that TRIM59 protein level was significantly higher in all of NSCLC cell lines that were examined than that in HBE (Fig 1E). Taken together, these results suggest that TRIM59 is overexpressed in NSCLC cell lines and may involve in the regulation of NSCLC growth.

### TRIM59 promotes the proliferation of NSCLC cells

**Fig 2:**
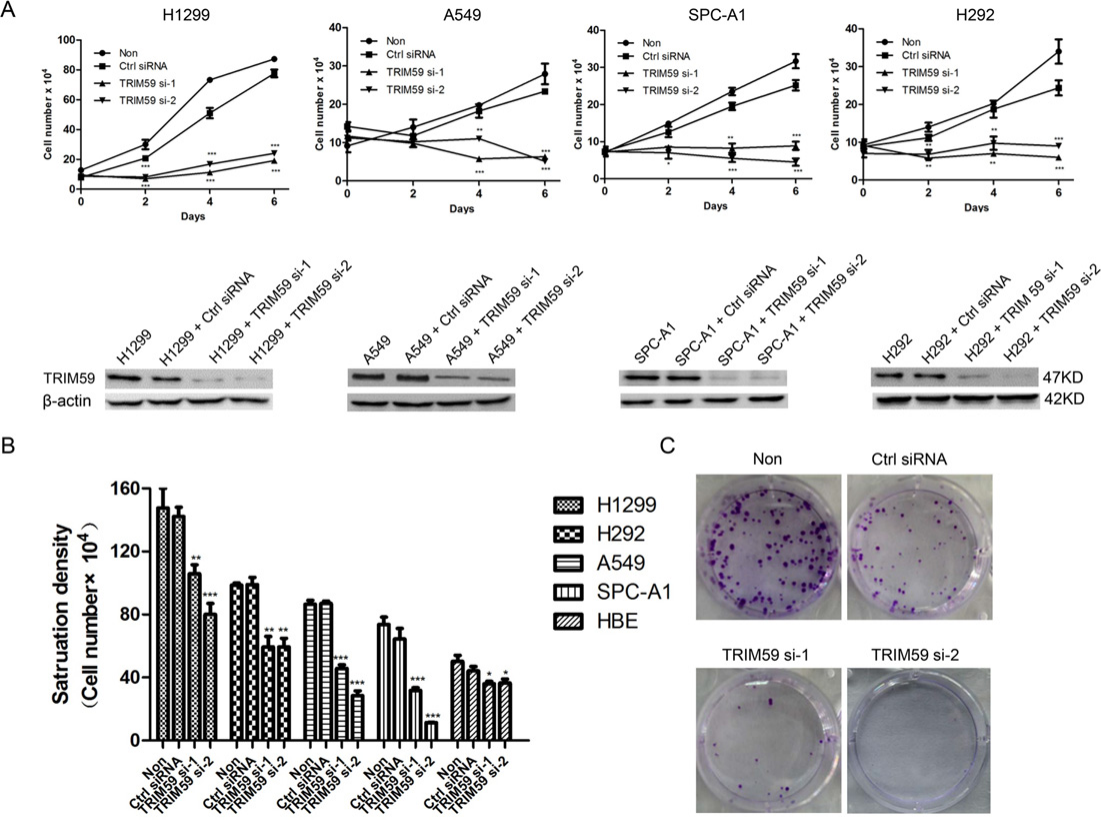
TRIM59 promotes the proliferation of NSCLC cells. **(A)** Low serum assay. The indicated NSCLC cell lines transiently transfected with TRIM59 siRNA-1, TRIM59 siRNA-2, control siRNA or untransfected were cultured in RPMI 1640 medium supplemented with 1% FBS. At the indicated times, cells were trypsinized and counted. The data represent the average of three independent experiments (mean±SD) (Top figures). Immunoblot of TRIM59 to check the knockdown efficiency of TRIM59 siRNAs in the indicated cell lines (Bottom figures). **(B)** Saturation density assay. The indicated NSCLC cells transiently transfected with TRIM59 siRNA-1, TRIM59 siRNA-2, control siRNA or untransfected were cultured in RPMI 1640 supplemented with 10% FBS for 6 days, trypsinized and counted. The data represent the average of three independent experiments (mean±SD). **(C)** Colony formation assay. Five hundred H1299 cells transiently transfected with TRIM59 siRNA-1, TRIM59 siRNA-2, control siRNA or untransfected were seeded in 6-well plates in RPMI 1640 with 5% FBS. After two weeks, cells were fixed and stained with crystal violet. Representative wells were photographed and shown.

To examine the function of TRIM59 in NSCLC cells, we purchased three siRNAs that target to human TRIM59 from Life Technologies Company. These siRNAs were transfected into H1299 cell line. After 48 hours, the protein level of TRIM59 was detected by western blot. As shown in the bottom figures of Fig 2A, both siRNA-1 and siRNA-2 efficiently knocked down TRIM59 in different cell lines compared with that of control siRNA. Next, we examined the effects of siRNA-induced knocking down of TRIM59 on the low serum growth of NSCLC cell lines. As shown in the top figures of Fig 2A, all NSCLC cells were extremely effective at growing in low serum, however when TRIM59 was knocked down, they were unable to grow under these conditions.

Similar results were obtained when examining the relative ability of TRIM59 to enable NSCLC cells to increase their saturation density. The cells treated with TRIM59 siRNA were significantly less dense than that treated with control siRNA or untreated. Similarly, siRNA-induced knocking down of TRIM59 significantly retarded the growth of NSCLC cells, but not significantly affected the growth of HBE cells (Fig 2B). Next, we performed the colony formation experiments. As shown in Fig 2C, the knocking down of TRIM59 dramatically decreased the colony formation in H1299 cells. Taken together, these results suggest that TRIM59 is essential for proliferation, colony formation of NSCLC cell lines. A recent study suggested that TRIM59 promotes gastric tumor growth at least partially through p53 [13]. As H1299 cells is a p53 deletion cell line (ATCC information), our results suggested that TRIM59 may target other proteins to promote cell growth of NSCLC.

### TRIM59 promotes the migration of NSCLC cells

**Fig 3:**
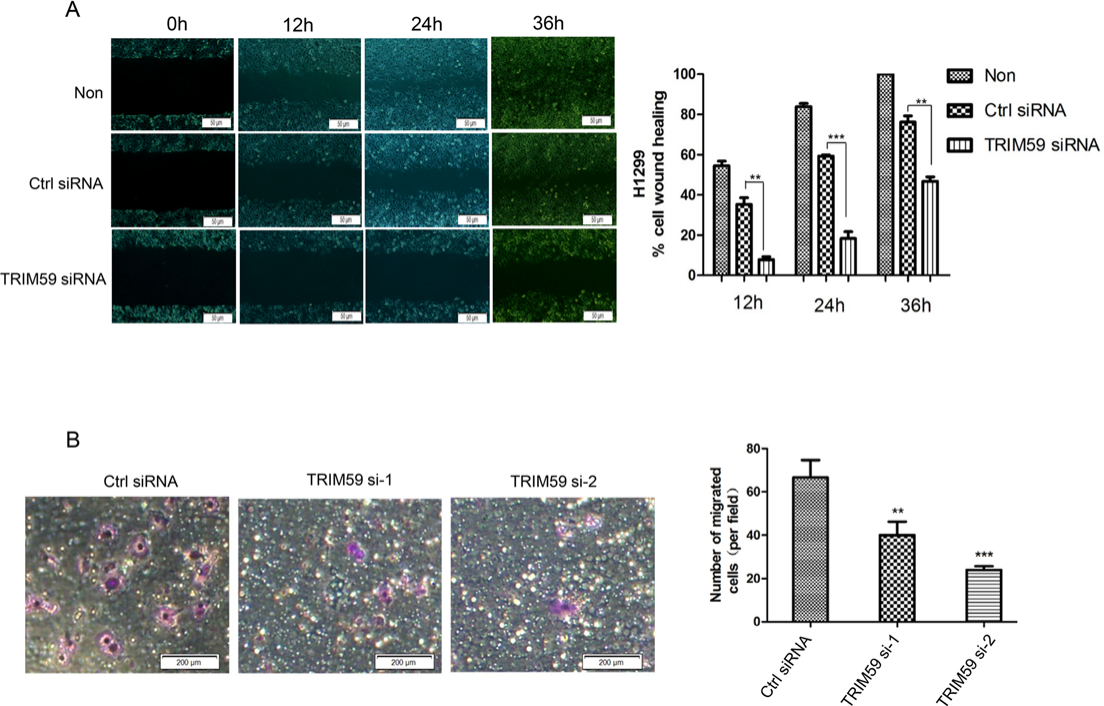
TRIM59 promotes the migration of NSCLC cells. **(A)** H1299 cells transiently transfected with TRIM59 siRNA-1, control siRNA or untransfected were cultured to create a confluent monolayer of 90–100% confluence, then the monolayer was scraped in a straight line to create a “scratch”. The extent of cell migration was photographed at the indicated times (Left figures). The transverse scratch wounds were re-examined and analyzed using image J software at 3 different sites from each wound area of gaps at each time point. Results are presented as mean ± standard error (Right figure). (B) H1299 cells transiently transfected with control siRNA or TRIM59 siRNA-1 or TRIM59 siRNA-2 and cultured with RPMI 1640 containing 10% FBS for 48hrs. Then cells were trypsinized and seeded in transwell chambers. After incubation for 10hrs, cells were fixed, stained, photographed and counted in five random views.

Given the ability of TRIM59 to promote NSCLC cell growth and colony formation, we are interested in examining its potential effects on NSCLC cell migration. To explore these possible effects, wound healing assay was performed. The results showed that H1299 cells migrated and the open area created by the “wound” was almost healed after 36 hours. However, the healing of the open area was markedly attenuated when TRIM59 was knocked down (Fig 3A). In order to further prove this effects, we also did transwell assay, as shown in Fig 3B, the number of migrated cells was greatly reduced when TRIM59 was knocked down. Therefore, knocking down TRIM59 hindered H1299 cell migration.

### Knocking down of TRIM59 arrests NSCLC cell cycle in G2 phase

**Fig 4:**
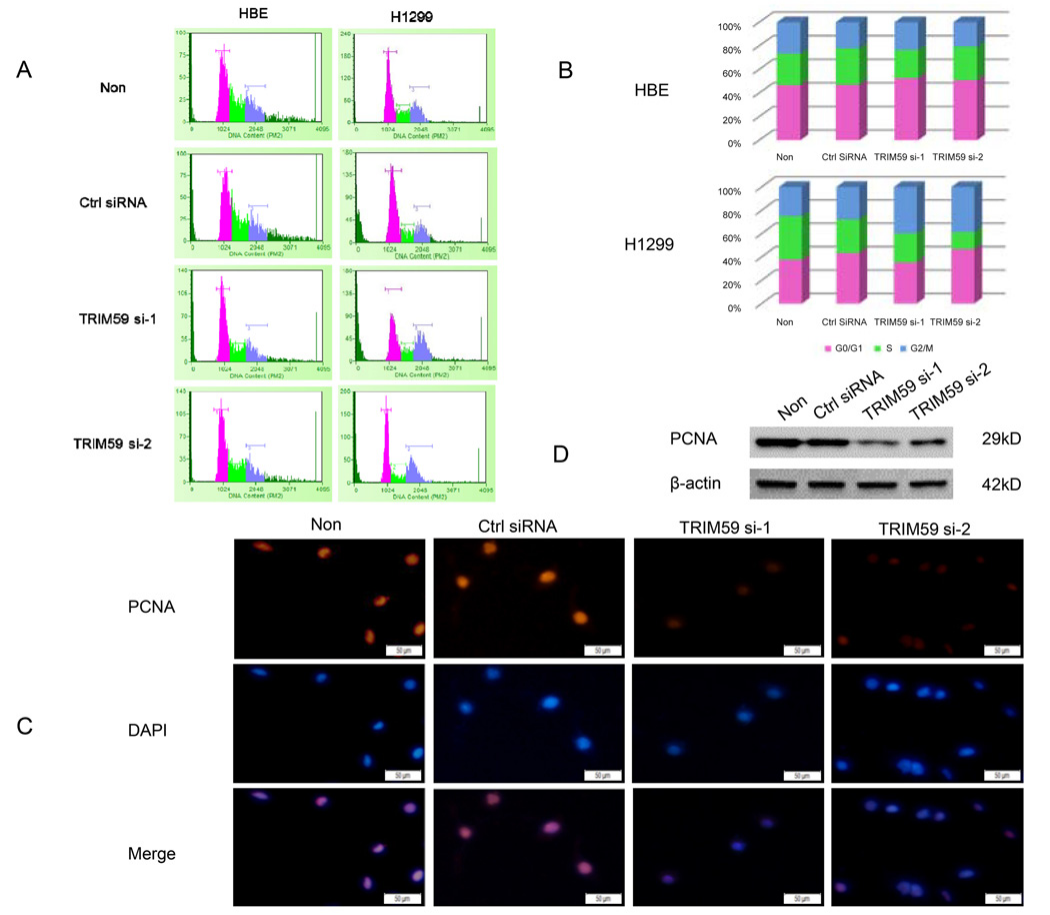
Knocking down of TRIM59 arrests NSCLC cell cycle in G2 phase. HBE and H1299 cells were transiently transfected with TRIM59 siRNA-1, TRIM59 siRNA-2, control siRNA or untransfected were cultured in RPMI 1640 with 10% FBS for 48 hrs. **(A)** Adherent cells were collected and cell cycle analysis was done by flow cytometry. The inserts showed the proportion of cells for each phase and are marked with different colors (pink: G0/G1 phase, green: S phase, and gray: G2/M phase). **(B)** The ratio of the cells in each phase was counted. **(C)** H1299 cells were fixed and stained with anti-PCNA antibody (red) and DAPI (blue). **(D)**The protein expression levels of PCNA in H1299 cells were checked by western blot.

Knocking down TRIM59 inhibited NSCLC cell growth indicating that TRIM59 may perturb cell cycle-related events in NSCLC cells. To examine the effects of TRIM59 knocking down on cell cycle, we transfected TRIM59 siRNA or control siRNA into HBE or H1299 cells, cell cycle was analyzed by flow cytometry after propidium iodide staining. As shown in Fig 4A and 4B, knocking down of TRIM59 significantly increased the proportion of G2/M phase and decreased the proportion of S or G0/G1 phases compared with that of control siRNA treated or untreated H1299 cells. To more precisely define TRIM59 knocking down arrest the cell cycle of H1299 whether in G2 or M phase, we next examined the PCNA by immunofluorescence staining and western blot. As shown in Fig 4C, we found that knocking down TRIM59 in H1299 cells dramatically down-regulated the staining of PCNA. Morever, western blot result also showed that PCNA protein level was down-regulated (Fig 4D). Taken together, these results suggested that the decreased cell proliferative activity in TRIM59 knocking down cells were caused by arresting cell cycle in G2 phase. Interestingly, knocking down of TRIM59 has no obvious effect on the cell cycle of HBE cells (Fig 4A and 4B), this could explain why knocking down of TRIM59 did not have significant inhibitory effects on the growth of HBE cells because had no effects on cell cycle.

### Knocking down of TRIM59 decreases the expression of cell cycle proteins

**Fig 5:**
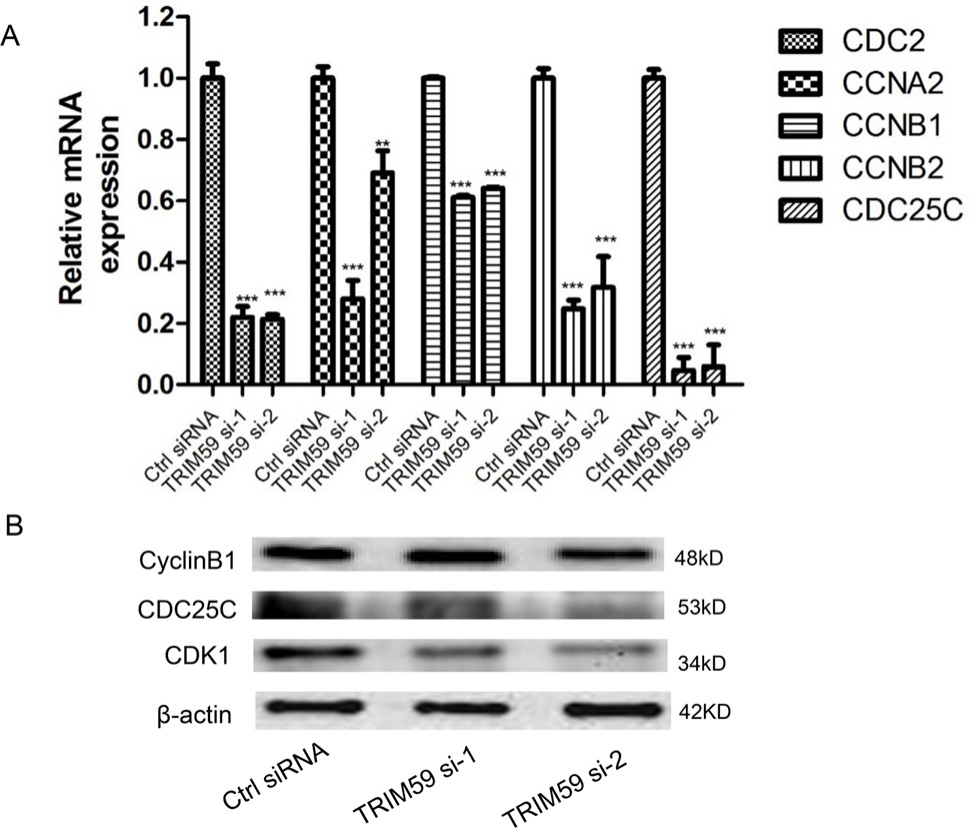
Knocking down of TRIM59 decreased the expression of cell cycle proteins. H1299 cells transiently transfected with TRIM59 siRNA-1, TRIM59 siRNA-2 or control siRNA were cultured in RPMI 1640 with 10% FBS for 48 hrs. **(A)** The mRNA expression levels of G2/M phase related genes CDC2, CCNA1, CCNB1, CCNB2 and CDC25C were tested by quantitative Real-Time PCR. **(B)** The protein expression levels of CDK1 (also known as CDC2), CDC25C and cyclin B1 were checked by western blot with indicated antibodies.

To elucidate the mechanism of cell cycle arrested by TRIM59 knocking down, we performed Q-PCR to check the mRNA levels of multiple genes which play essential roles in G2/M phase. As shown in Fig 5A, the mRNA levels of CDC2, CCNA1, CCNB1, CCNB2 and CDC25C were significantly reduced in TRIM59 siRNA treated cells compared with that in control siRNA treated cells. Furthermore, we examined the protein levels of cyclin B1, CDC25C and CDK1 by western blot. As shown in Fig 5B, CDC25C and CDK1 but not cyclin B1 was significantly decreased in TRIM59 siRNA treated cells compared with that of control siRNA treated cells. These findings together suggest that TRIM59 is important for cell cycle regulation by affecting cell cycle proteins.

### TRIM 59 promotes NSCLC cell growth not through p53 signaling pathway

**Fig 6:**
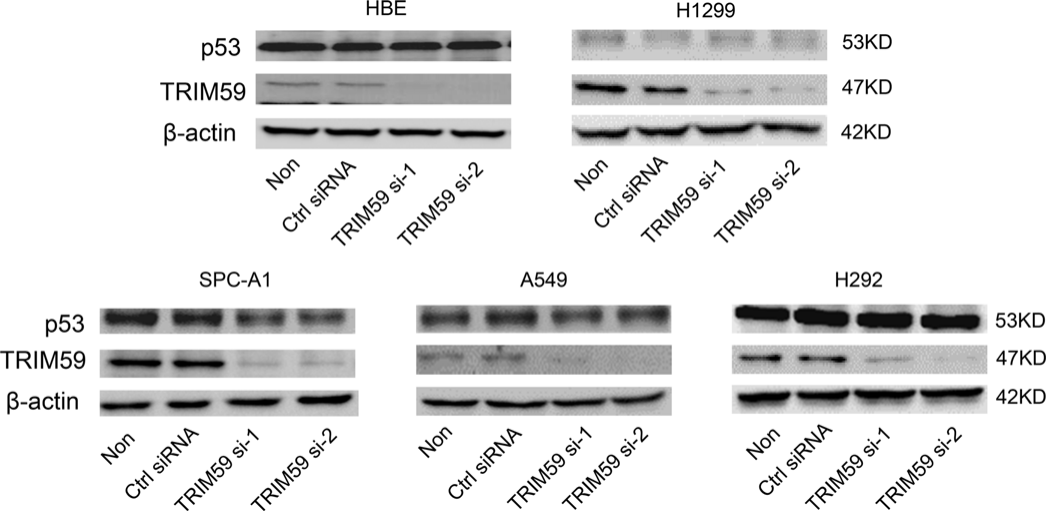
TRIM59 promotes NSCLC cell growth not through p53 signaling pathway. Four types of indicated NSCLC cells and HBE cells transiently transfected with TRIM59 siRNA-1, TRIM59 siRNA-2, control siRNA or untransfected were cultured in RPMI 1640 with 10% FBS for 48 hrs. The protein expression levels of p53 were checked by western blot using anti-p53 and anti-TRIM59 antibodies.

Zhou et al (2014) reported that in gastric tumor, TRIM59 interacts with p53, promoting its ubiquitination and degradation; TRIM59 might promote gastric carcinogenesis via this mechanism [13]. To investigate the mechanism of TRIM59 promoting cell growth in NSCLC cells, we knocked down TRIM59 and checked the protein levels of p53 in NSCLC cell lines and HBE cells. For H1299 cells, it is a p53 deletion cell line, p53 protein could not be detected. Intriguingly, for HBE, H292 and A549 cells, they are cell lines for p53 wild type [17], the p53 protein levels were not affected by TRIM59 knocking down. However, for SPC-A1 cells, p53 protein levels were decreased when TRIM59 was knocked down (Fig 6). Taken together, we proposed that TRIM59 may promote NSCLC cell growth through other pathways but not the p53 signaling pathway.

## Discussion and Conclusions

TRIM proteins comprise a large superfamily and are important regulators of cellular processes including inflammation, immunity, cell proliferation and apoptosis, and antiviral [1, 4–6, 10]. The objective of this study is to identify the TRIM proteins that are potentially involved in regulating proliferation, colony formation and migration of NSCLC cells. We used Q-PCR to profile the expression levels of 72 TRIM genes in four NSCLC cell lines and a normal human bronchial epithelial (HBE) cell line. We found that the expression levels of 10 TRIM genes including TRIM3, TRIM7, TRIM14, TRIM16, TRIM21, TRIM22, TRIM29, TRIM59, TRIM66 and TRIM70 were significantly upregulated in NSCLC cells compared with that in HBE cells, whereas the expression levels of other 7 TRIM genes including TRIM4, TRIM9, TRIM36, TRIM46, TRIM54, TRIM67 and TRIM76 were significantly down-regulated in NSCLC cells compared with that in HBE cells. Although the most of TRIM proteins are less characterized, some TRIM proteins play important roles in the cellular processes related to cancer. For example, TRIM3, TRIM16, TRIM29 and TRIM36 have been reported to function as tumor suppressors [18–21]. Interestingly, TRIM7 was identified as glycogenin-interacting protein and involved in the regulation of cellular glucose metabolism [22]. As the unique feature in cancer cell metabolism is essential to the growth and migration of cancer cells, it would be important to further investigate the role of TRIM7 in the cellular metabolism of NSCLC cells. In addition, TRIM67 has been reported to function as a Ras inhibitor [23]. The down regulation of TRIM67 in NSCLC cells may contribute to the over-activation of Ras signaling and over growth of NSCLC cells.

We further characterized the biological function of TRIM59 in the proliferation, colony formation and migration of NSCLC cells. SiRNA-induced knocking down of TRIM59 significantly attenuated the growth, colony formation and migration of NSCLC cells, but not significantly affected the growth of HBE cells. However, in vivo experiments need be performed to verify these results. Khatamianfar et al. recently identified TRIM59 as an early signal transducer in two (SV40Tag and Ras) oncogene pathways in murine prostate cancer models. In addition, Zhou et al. also reported that TRIM59 promoted gastric tumorigenesis through down-regulation of p53 protein abundance and suppression of p53 downstream signals. To check whether TRIM59 promotes the proliferation of NSCLC cells also through p53 pathway, we knocked down TRIM59 and checked p53 expression. For H1299 cell, it is a p53 deletion cell line, for other cell lines, no effects or even p53 decreased. So TRIM59 regulates the proliferation and migration of NSCLC cells by targeting other proteins than p53.

In conclusion, we have identified that TRIM59 is a potential important regulator for the proliferation and migration of NSCLC cells. SiRNA-induced knocking down of TRIM59 significantly inhibited the proliferation and migration of NSCLC cell lines by arresting cell cycle in G2 phase. Furthermore, TRIM59 knocking down affected the expression of a number of cell cycle proteins including CDC25C and CDK1. However, the in vivo physiological role and mechanisms of TRIM59 protein need to be further investigated.

## Supporting Information

S1 Fig The mRNA expression levels of TRIM genes including TRIM4, TRIM9, TRIM36, TRIM46, TRIM54, TRIM67 and TRIM76 were significantly down-regulated in NSCLC cell lines compared with that in HBE cells. (TIF)

S1 Table The profile for expression patterns of TRIM gene family in NSCLC cell lines. We selected four NSCLC cell lines including H1299, SPC-A1, A549 and H292. The human bronchial epithelial cell (HBE) was served as the control. The mRNA levels of TRIM gene family in these cell lines were measured by Q-PCR. The mRNA expression level of TRIM genes with cycle times ≥ 34 were determined to be undetected. TRIM17, TRIM40, TRIM42, TRIM43, TRIM48, TRIM49, TRIM50, TRIM51, TRIM63, TRIM60, TRIM64 and TRIM77 were undetected. Mean: average; SD: standard error; CT: cycle time.
(XLSX)
