# Supplementary figures and images for: TRIM59 Promotes the Proliferation and Migration of Non-Small Cell Lung Cancer Cells by Upregulating Cell Cycle Related Proteins
Source: PLoS One. 2015 Nov 24;10(11):e0142596. doi: 10.1371/journal.pone.0142596 (PMC4658198; doi:10.1371/journal.pone.0142596)

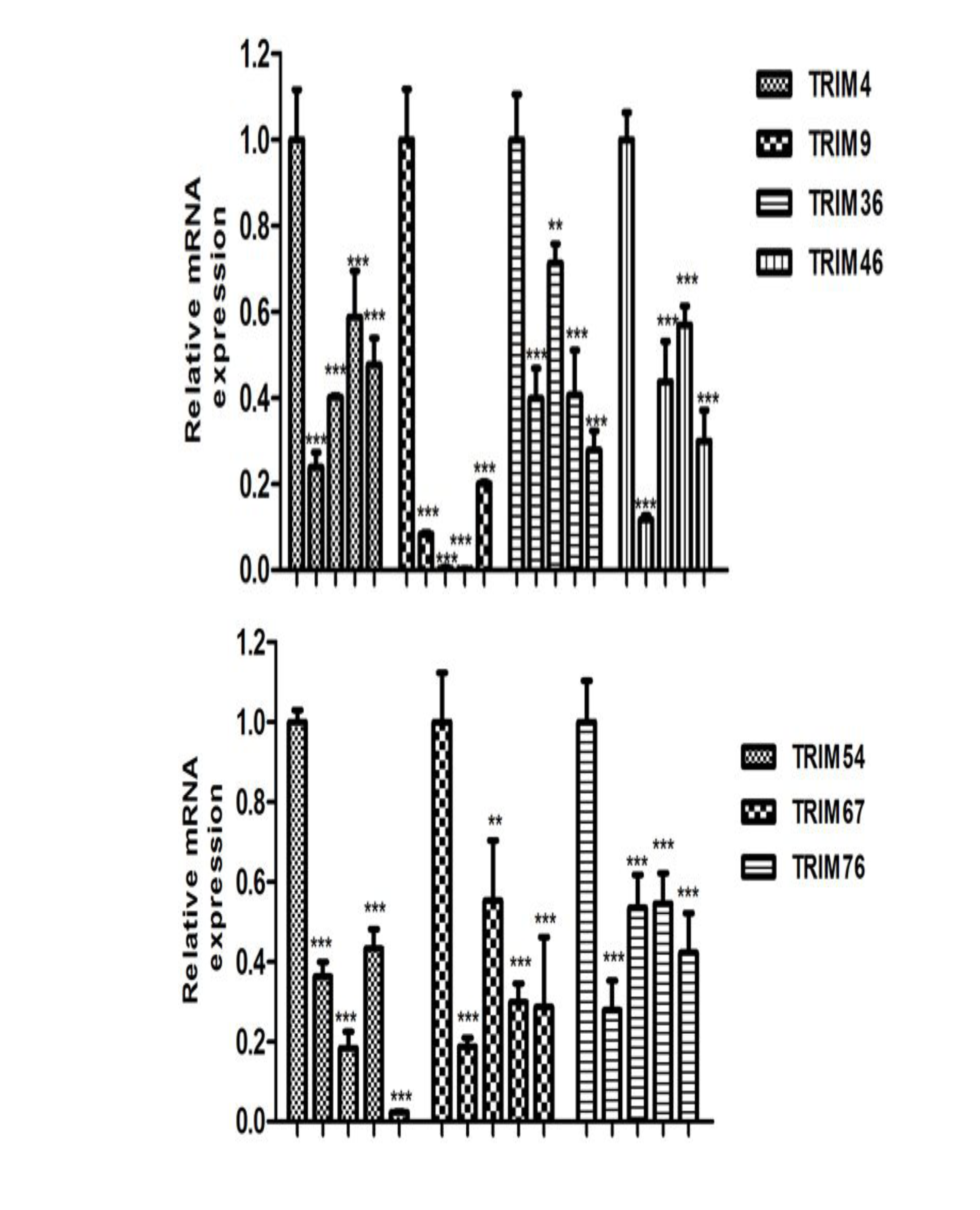

Supplement: S1 Fig — (TIF) [file pone.0142596.s001.tif]
